# Supplementary material for: Aged gut microbiota contribute to different changes in antioxidant defense in the heart and liver after transfer to germ-free mice
Source: PLoS One. 2023 Aug 11;18(8):e0289892. doi: 10.1371/journal.pone.0289892 (PMC10420372; doi:10.1371/journal.pone.0289892)
Supplement: Attachment — Submitted filename: Response to editor 719.docx [file pone.0289892.s001.docx]

**Response to Editor (s)**

Dear editor (s),

Thank you very much for your attendance on our manuscript (PONE-D-23-09106) entitled “Aged Gut Microbiota Contribute to Different Changes in Antioxidant Defense in the Heart and Liver after Transfer to Germ-free Mice”.

It is very pleasure for us to revise our manuscript required. We have seriously revised our manuscript according to the reviewers by red color. And we also revised our manuscript according to suggestions given by you; the detail changes are as follows.

1. **Please ensure that your manuscript meets PLOS ONE's style requirements, including those for file naming.**

**Answer:** Thank you very much for your kind suggestion. We have revised our manuscript according to PLOS ONE's style requirements, including those for file naming.

2. **We suggest you thoroughly copyedit your manuscript for language usage, spelling, and grammar. If you do not know anyone who can help you do this, you may wish to consider employing a professional scientific editing service.**

**Answer:** Thank you very much for your kind suggestion. Our manuscript has been checked by American Journal Experts (AJE). The editing certificate and the manuscript showing changes (supporting information) have been uploaded.

3. **We note that the grant information you provided in the ‘Funding Information’ and ‘Financial Disclosure’ sections do not match.**

**Answer**: Thank you very much for your kind review. We are very sorry for the inconsistent of information between ‘Funding Information’ and ‘Financial Disclosure’. The final funding information of this study is as follows.

“**Source of research financing**:

This research was financially supported by the Sichuan Provincial Science and Technology Plan Project (2022YFS0432), Basic Research Project of Sichuan Province (2022JDKY0013), Scientific and Technical Research Project of Sichuan Provincial Administration of TCM (2023MS485).”

4. **Thank you for stating the following financial disclosure:**

**Answer**: Thank you very much for your kind comments. The financial disclosure is listed as follows.

“The funders had no role in study design, data collection and analysis, decision to publish, or preparation of the manuscript.”

5. **We note that you have provided funding information that is not currently declared in your Funding Statement. However, funding information should not appear in the Acknowledgments section or other areas of your manuscript. We will only publish funding information present in the Funding Statement section of the online submission form.  Please remove any funding-related text from the manuscript and let us know how you would like to update your Funding Statement.**

**Answer:** Thank you very much for your kind review. We have removed the funding-related text from the manuscript.

6. **PLOS requires an ORCID iD for the corresponding author in Editorial Manager on papers submitted after December 6th, 2016. Please ensure that you have an ORCID iD and that it is validated in Editorial Manager.**

**Answer:** Thank you very much for your kind suggestion. According to your instructions, we have made a link of ORCID ID in Editorial Manager.

7. **Your ethics statement should only appear in the Methods section of your manuscript. If your ethics statement is written in any section besides the Methods, please move it to the Methods section and delete it from any other section. Please ensure that your ethics statement is included in your manuscript, as the ethics statement entered into the online submission form will not be published alongside your manuscript.**

**Answer:** Thank you very much for your kind suggestion. According to your comment, the ethics statement has been moved to Methods section.

If you need any more information or having some suggestion referring this manuscript, please don’t hesitate to contact us. Any of your suggestion would do great help on our manuscript. Thanks again.

Your sincerely

                                                                                                                                                                                                                                                Lei Zhang
